# Chromium-Induced Reactive Oxygen Species Accumulation by Altering the Enzymatic Antioxidant System and Associated Cytotoxic, Genotoxic, Ultrastructural, and Photosynthetic Changes in Plants

**DOI:** 10.3390/ijms21030728

**Published:** 2020-01-22

**Authors:** Abdul Wakeel, Ming Xu, Yinbo Gan

**Affiliations:** 1 Key Laboratory of Geospatial Technology for the Middle and Lower Yellow River Regions, College of Environment and Planning, Henan University, Kaifeng 475004, China; awzju@yahoo.com; 2 Zhejiang Key Lab of Crop Germplasm, Department of Agronomy, College of Agriculture and Biotechnology, Zhejiang University, Hangzhou 310058, China

**Keywords:** reactive oxygen species, antioxidants, cytotoxicity, genotoxicity, photosynthesis

## Abstract

Chromium (Cr) is one of the top seven toxic heavy metals, being ranked 21st among the abundantly found metals in the earth’s crust. A huge amount of Cr releases from various industries and Cr mines, which is accumulating in the agricultural land, is significantly reducing the crop development, growth, and yield. Chromium mediates phytotoxicity either by direct interaction with different plant parts and metabolic pathways or it generates internal stress by inducing the accumulation of reactive oxygen species (ROS). Thus, the role of Cr-induced ROS in the phytotoxicity is very important. In the current study, we reviewed the most recent publications regarding Cr-induced ROS, Cr-induced alteration in the enzymatic antioxidant system, Cr-induced lipid peroxidation and cell membrane damage, Cr-induced DNA damage and genotoxicity, Cr-induced ultrastructural changes in cell and subcellular level, and Cr-induced alterations in photosynthesis and photosynthetic apparatus. Taken together, we conclude that Cr-induced ROS and the suppression of the enzymatic antioxidant system actually mediate Cr-induced cytotoxic, genotoxic, ultrastructural, and photosynthetic changes in plants.

## 1. Introduction

Chromium (Cr), heavy metal with a range of oxidation numbers [Cr(II) to Cr(VI)], which is placed in the group (VI-B) of transition elements in the modern periodic table [1]. Chromium, which is the hard silver color metal with 7.19 g/cm^3^ density, 51.10 g/M molecular weight, and 24 atomic number, has been ranked 21st among the most abundantly found metals on the earth’s crust [2]. The trivalent [chromite; Cr(III)] and the hexavalent [chromate; Cr(VI)] are the most stable naturally found Cr species [3]. Hexavalent form of Cr is a potentially strong oxidizing agent, and higher water solubility, mobility, and bioavailability make it the most toxic form of Cr as compared to other Cr species [4]. The oxygenated environment can convert Cr(III) into Cr(VI), the factors that are involved in maintaining the proper ratio of these Cr forms are oxygen concentration, pH, complexing factors, and reducing agents [5].

Chromium extraction from the mines has been excessively increased due to its increasing use in various industries [2]. Kazakhstan, South Africa, China, and India are the world-leading Cr using countries [2,6,7]. Leather tanning, metallurgy, electroplating, alloying, ceramic glazes, wood preservation, water corrosion inhibition, refractory bricks, pressure-treated lumber, textile dyes, and mordant, pigments and paints production, and paper and pulp production industries contribute to the hyperaccumulation of Cr in the environment. Furthermore, anthropogenic activities, such as the dumping Cr-contaminated liquids and solids wastes, are the reason for the hyperaccumulation of Cr in the environment [8,9,10,11]. The emission of Cr from the cooling towers of the industries and the dust rising from the roads and roadsides are considered to be the most important Cr sources [12,13].

Increased Cr accumulation in the agricultural land causes damage the plant growth and development at the organ, cellular, or even genetic level [14]. Cr-induced phytotoxicity is mostly mediated via induced reactive oxygen species (ROS), which cause the cellular and extracellular damage in plants [8]. In the current study, we reviewed Cr-induced ROS, associated cellular, and ultra-structural damages in plants

## 2. Chromium-Induced Oxidative Stress in Plants

Plants that are exposed to unfavorable conditions produce reactive oxygen species (ROS) as a defense mechanism [15,16]. The hyperaccumulation of ROS generates endogenous stress that can damage plant growth and development [8]. Hydrogen peroxide (H_2_O_2_), superoxide anion (O_2_^−^), singlet oxygen (^1^O_2_), hydroxyl ion (HO^−^), peroxyl (RO^−^), alkoxyl (RO^−^), and organic hydroperoxide (ROOH) are the various ROS that are found in plants [2,17,18]. Reactive oxygen species are produced in the mitochondria, peroxisome, and chloroplast as a byproduct of various biochemical reactions [18,19,20,21]. Plants mechanisms that are in the regulation of ROS level include ROS biosynthesis, enzymatic, and/or non-enzymatic ROS scavenging [8]. Heavy metals, such as lead (Pb), cadmium (Cd), aluminum (Al), nickel (Ni), and Cr, are reported for the enhancement in ROS productions and accumulation [8,19,22]. Various plant species that are exposed to toxic Cr level or industrial wastes containing the toxic level of Cr, showed induced ROS accumulation, as summarized in Table 1.

Chromium-induced ROS accumulation mediates various physiological, biochemical, molecular, and developmental changes in plants [41]. These alterations in the physiological and biochemical process may be provoked by directly interacting with enzymes, lipids, proteins, and genetic material (DNA and/or RNA), or by Cr-induced ROS accumulation [8,50,51]. Cr direct interaction or Cr-induced ROS both mediated membrane damage, degradation and deactivation of genetic material, proteins, and enzymes, which resulted in the growth inhibition by the suppression cell division or activation programmed cell death [8,52,53].

**Figure 1:**
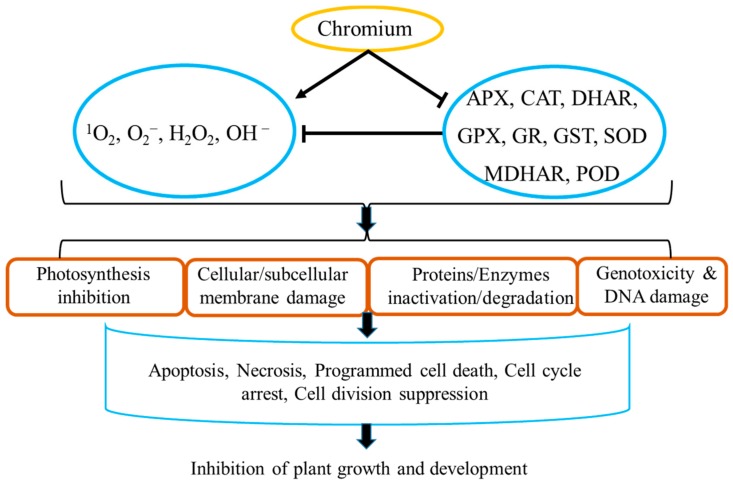
Cr(VI)-induced ROS mediated alteration in plants: Cr(VI)-induces ROS accumulation by suppressing enzymatic antioxidant system, which damages cellular and subcellular membranes; induces ultrastructural changes in cell organelles such as mitochondria, plastids, and thylakoids; inhibits protein and enzymes at transcriptional or post-transcriptional level as well as degrades various enzymes and proteins; and DNA damages. All of these alterations inhibit photosynthesis and trigger and enhance necrosis, apoptosis, and programmed cell death, and significantly inhibit plant growth and development. Superoxide (O^2−^), hydrogen peroxide (H_2_O_2_), hydroxyl ion (HO^−^), and singlet oxygen (^1^O_2_). Ascorbate peroxidase (APX), catalase (CAT, dehydroascorbate reductase (DHAR), glutathione peroxidase (GPX), glutathione reductase (GR), glutathione S-transferase (GST), monodehydroascorbate reductase (MDHAR), peroxidase (POD), and superoxide dismutase (SOD). T-bars represent inhibition or suppression of the target, arrows represent promotion or upregulation of the target, and bold arrows represent the ultimate downstream result or impact of the process.

Chromium-induced ROS mediates ultra-structural alteration in various plant tissues and irreversibly degrades biomolecules, except for DNA, cysteine, and methionine, which can be restored, in a dose-dependent and tissue-specific manner [23,45,49,54]. Reactive oxygen species are produced during the reduction reaction of Cr(VI) to Cr(III) and Fenton reaction. The catalytic power of Cr(III) is greater than iron (Fe), copper (Cu), cobalt (Co), manganese (Mn), and zinc (Zn) in the Fenton reaction [2,45,54,55]. The Cr involvement in such reactions is not well studied and some other intermediates and factors may also be involved in the Cr-induced ROS generation [8]. ROS mediated various physiological, biochemical, molecular, and ultrastructural changes, as shown in Figure 1.

## 3. Chromium-Mediated Alteration in the Enzymatic Antioxidant System

Plants have developed a complex and well-organized enzymatic antioxidant system to deal with access ROS, produced by various endogenous and exogenous stimuli, including toxic Cr levels [8]. Superoxide (O_2_^−^) is converted to H_2_O_2_ by superoxide dismutase (SOD). H_2_O_2_ is converted by ascorbate peroxidase (APX), peroxidase (POD), and catalase (CAT) to H_2_O [8,56]. Furthermore, to minimize the Cr, cadmium (Cd), bisphenol A (BPA), and other abiotic stresses mediated oxidative stress, plants use the enzymatic antioxidant system, which includes, SOD, APX, POD, CAT, glutathione reductase (GR), monodehydroascorbate reductase (MDHAR), dehydroascorbate reductase (DHAR), glutathione peroxidase (GPX), and glutathione S-transferase (GST) [8,17,21,50,57,58]. Previous studies have reported that Cr-induces the alteration in the production and accumulation of enzymatic antioxidant system for the regulation and scavenging Cr-induced ROS have been summarized in Table 2.

## 4. Chromium-Induced Lipid Peroxidation

Lipid peroxidation is initiated by the increased ROS accumulation through the decomposition of membrane lipids and proteins, and it is one of the primary reasons for abiotic stress-induced cell damages [82]. Chromium stress has been reported for the induced ROS production, and it has been also reported for biological membrane damage [2]. One of the lipid peroxidation products, called malondialdehyde (MDA), which is considered as an oxidative damage indicator, has been greatly studied in the heavy metals mediated damage of biological membrane, including Cr [8,82]. Chromium-induced ROS mediated lipid peroxidation in various plant species, including economically important crops, has been summarized in Table 3.

## 5. Chromium-INDUCED DNA DAMAGE and Genotoxicity

Genotoxicity is one of the most serious threats of heavy metals toxicity to living organisms [101,102]. DNA damage can have serious consequences, such as deregulation or mutagenesis of the cell replication process, leading to tumor formation, and ultimately cell death [101,103,104]. Heavy metals cause DNA damage either by direct interaction with DNA or by induced ROS accumulation, which is considered to be one of the main internal causes of DNA damage (Figure 1). Heavy metals not only induce DNA damage, but also interrupt DNA damage repair mechanisms [101,102].

In contrast to other heavy metals, which are directly interacting with DNA, Cr-induces ROS mediated genotoxicity [105]. Chromium-induced genotoxicity and carcinogenic effects are greatly investigated in yeast and animal cells. Its carcinogenic effects have been also reported in the workers, working in the Cr mines and Cr consuming industries [101,104,105,106]. In vivo and in vitro investigations revealed that Cr(VI) produces various types of structural alterations in genetic materials, including inter-DNA strand cross-links, DNA chromosomal protein cross-links, and nucleotide strand breaks [105,107,108].

Chromium-DNA adducts (association of Cr with phosphodiester backbone of DNA), which are mainly reported in mammalian cells, being considered the primary cause of Cr(VI) induced mutagenicity [105,109]. Cr(VI)-mediated genotoxicity has been reported in humans, rats, fish, fish cell lines, yeast, and bacteria [105,108,110,111,112,113]. Some studies have reported that Cr(III) is also interacting with DNA to form a covalent bond with the phosphate backbone [105]. Cr(III) also interacts with the DNA base pairs’ stacking mode, which leads to DNA lesion, cleavage, and the DNA single/double-strand breakage [105,108]. Cr(VI)-induced ROS mediates these various DNA degradations [45]. The current study reviews chromium-induced chromosomal fragmentation and bridging, alteration in DNA methylation, DNA mutation, increase in percent tail DNA, tail moment, and percent DNA damage in tail length, chromosome aberrations or micronuclei formations, DNA inter/intrastrand crosslinks, protein-DNA crosslinks, DNA-single/double-strand breaks, DNA adducts, DNA transcription, and replication dysfunction, abnormal DNA repair mechanisms, changes in signaling pathways for survival, genomic instability, oxidized bases, instability of microsatellites, and genetic/epigenetic alteration in different plant species t (Table 4).

## 6. Chromium-Induced Ultrastructural Changes

### 6.1. Cr-Induced Necrosis and Cellular Injury

Chromium-induced cytotoxicity affects essential micronutrient absorption, lipid peroxidation, cell cycle arrest and ultimate cell death in plants [8,22,56,87]. Toxic Cr levels also mediate the stomatal abnormalities, such as the decreased size of stomatal aperture, swelling of guard cells, changes in membrane permeability level, ion flux, and osmotic pressure [22,87,114,115]. These stomatal aberrations significantly influence the a, b, and total chlorophyll contents, stomatal conductance, photosynthetic rate, respiration, and transpiration rate [87,114,115]. Trichomes, which are unicellular outgrowths on the leaf, play a defensive role in plants under stress conditions [116,117]. Metal ions’ active transport regulates the number and distribution of trichomes, and an increased trichome number has been noticed in the plants exposed to toxic Cr(VI) levels [22].

Exposure to high Cr-concentration causes mitochondrial damages, such as outer membrane rupture, swelling, deformed or altered internal cristae, dense electron accumulated materials, and spherical morphology [23,118,119]. It has been also reported that mitochondria were underdeveloped in the *Brassica napus* seedlings that were exposed to 400 μM Cr as compared seedlings exposed to control conditions [41,120]. The ultrastructural investigations also revealed that Cr(VI) stress alters plastid structure, more specifically, chloroplast, with a spherical and contracted morphology [120,121,122,123]. The irregular shape and size of the chloroplast with contained large plastoglobuli and starch grains were reported in *Spirodela poyrhiza* seedlings that were exposed to high Cr(VI)-level [23]. Cell membrane injury, disruption of cytoplasm, and vacuole upon Cr exposure are frequently reported [23,120,121]. Table 5 summarizes the ultrastructural changes reported in the different plant species exposed to Cr-stress.

### 6.2. Electron-Dense Material Deposition in the Subcellular Compartments

Plants restrict the accumulation of heavy metals in the less sensitive organelles to avoid damage to the more sensitive organelles at the cellular level [16,142,143]. The precipitation of electron-dense granules in subcellular compartments, especially in the cell wall, is the first line cellular defense mechanism, against toxic heavy metals [23,144,145]. The electron-dense deposition in the interspace between the cell wall and cell membrane, vacuoles, plastids, between the cisternae of endoplasmic reticulum, and cytoplasm in the seedlings of *Arabidopsis* that were exposed to Cr(VI) have been previously reported [23,136]. The deposition of electron-dense material in the pectic middle lamella instead of cellulosic/hemicellulosic components of *Arabidopsis* root tip cells has been also reported [23].

There is a prominent difference in the degree of Cr(VI)-induced damages among the different cellular compartments of plants [23,47,136]. The cellular compartments, such as mitochondria, plastids, Golgi bodies, and vacuoles, were severe; cytoplasm, cell membrane, endoplasmic reticulum (ER) were mild; cell wall and nuclei were moderately damaged in the seedlings of *Arabidopsis* that were exposed to high Cr(VI) levels [23], as shown in Table 5.

## 7. Chromium-Mediated Changes in Photosynthesis and Photosynthetic Apparatus

Various heavy metals that influence plant biochemical, physiological, and metabolic processes affect photosynthesis and photosynthetic apparatus, leading to reduced plant growth and yield [3,8,19,23]. The effect of Cr on the photosynthesis and photosynthetic apparatus has been greatly studied in different plant species, and it mainly influences the enzymatic activities, electron transport chain, CO_2_ fixation, photosynthetic phosphorylation, and structure of plastids [35,65,146,147]. In various plant species Cr-reduced chlorophyll contents, carotenoids, and photosynthetic activities have been greatly investigated, as summarized in Table 6. The structural changes in the chloroplast could be one of the factors that are involved in the defective photosynthesis [2]. Chromium-induced chloroplast ultrastructural changes mediate the suppression of photosynthesis in various plant species, as summarized in Table 5. Chromium-reduced alterations in the volume and auto-fluorescence of chloroplast [127], altered thylakoid arrangement, chloroplast membrane distortion, and negatively affected light/dark reactions have also been reported [2,22,96,148]. Electron transport chain inhibition might be due to the Cr-induced redox changes in the Fe and Cu carriers or binding of Cr to cytochrome groups to inhibit its oxidative activity [149,150,151].

Furthermore, high Cr-level mediates ROS accumulation, which is an alternative sink for the electron, being involved in the suppression of photosynthesis [8,127,152]. Heavy metals-induced ROS modulated alteration in the photosynthesis and photosynthetic machinery is intensely studied [60,76]. Destabilization and degradation of antenna complex proteins, Mg^+^ substitutions with H^+^ ion, and thylakoid membrane damage are the main steps in ROS assisted leaf pigment-protein structure and function retardation [2,153]. The Cr(VI)-induced degradation of a chlorophyll biosynthesis key enzyme delta-aminolaevulinic acid dehydratase, and its competing capability with Mg and Fe translocation to leaves are involved in the decreased photosynthetic pigments and photosynthesis [81,154]. High Cr-level in the soil greatly influences macro/micronutrient uptake. As Cr has no specific uptake channels, it is competing with essential elements for the uptake channels [155,156].

## 8. Strategies to Overcome Cr-Uptake and Phytotoxicity

Chromium (III) has an essential role in the human metabolic process [102]. However, none of the Cr species have been reported to be essential in plants, thus there is no specialized mechanism for Cr-uptake in plants [23]. In plants, Cr-uptake, which depends on the Cr-type and plant species, is carried out through essential nutrients uptake channels [167]. Plants uptake Cr(III) by passive mechanism, while the uptake of Cr(VI), which has a structural resemblance with sulfate and phosphate, takes palace by the active mechanism through sulfate and phosphate channels [2,28,167]. The restriction of Cr(VI)-uptake and no change in Cr(III)-uptake by the treatment of exogenous metabolic inhibitors confirmed the active and passive uptake mechanisms of these Cr species, respectively [2,89]. The molecular mechanism for Cr uptake and translocation is elusive and further studies are required.

Heavy metal ATPase (HMA), cation diffusion facilitator (CDF), superfamily of ATP binding cassette (ABC), natural resistance-associated macrophage protein (NRAMP), and ZRT IRT-like proteins (ZIP) are some of the gene families that are involved in the transportation of metals and heavy metals in plants [18]. Further investigations regarding the possible role of these gene families in Cr-uptake and translocation will increase our understanding of the Cr-transportation mechanism in plants. Some of the studies reported that Cr is sharing the iron, sulfate, and phosphate transport pathways in plants [55]. Thus, plants that are exposed to a toxic level of Cr-concentrations are also experiencing starvation of essential elements [168,169]. As Cr-competes with some essential metals for the uptake, these essential elements enriched environment can reduce Cr-uptake, transport, and toxicity in plants.

Iron enriched growth medium significantly reduced Cr(VI)-uptake and translocation in plants [170]. The pretreatment of seeds with salicylic acid, application of auxin and ethylene inhibitors to growth media, treatment of polyamine-brassinosteroid, 24-epibrassinolide, and plant growth-promoting bacteria reduce Cr-uptake, translocation, and toxicity [8,30,42,48,68,123,171]. The natural selection of Cr-tolerant varieties, conventional breeding, and targeted genes mutation can be used for the control of Cr-phytotoxicity and damage to yield of economically important crops.

## 9. Conclusions

Plants exposed to toxic Cr-level mediate high ROS accumulation by either oxidation and interconversion of one Cr form to other or by the inhibition enzymatic antioxidant system. Cr-induced ROS mediates DNA damage and genotoxicity, cytotoxicity, ultrastructural damages, and alteration in photosynthesis and photosynthetic apparatus. These alterations include necrosis, programmed cell death, cell cycle arrest, and suppression of cell division that ultimately reduce plant growth, development, and yield, as shown in Figure 1.

## Figures and Tables

**Table 1:** Accumulations and investigations of various ROS species in numerous plant species exposed to Cr(VI) and/or Cr(III). Superoxide (O_2_^−^), hydrogen peroxide (H_2_O_2),_ hydroxyl ion (HO^−^), and singlet oxygen (^1^O_2_).

Plant Species	Common Name	ROS Types	Cr(VI) Concentration	References
*Arabidopsis thaliana*	Arabidopsis	O_2_^−^, H_2_O_2_	100–400 µM	[8,23]
*Helianthus annuus*	Sunflower	O_2_^−^, OH^−^, H_2_O_2_	20 mg/L & 20 mg/Kg	[24,25,26]
*Zea mays*	Maize	O_2_^−^, H_2_O_2_, OH^−^	100–300 µM & 100–300 mg/Kg	[27,28,29,30,31,32]
*Brassica juncea*	Indian mustard	^1^O_2_, O_2_^−^, H_2_O_2_, OH^−^	300 µM	[17,33]
*Glycine max*	Soybean	H_2_O_2_	400 mg/kg & 500 mg/kg Cr(III)	[22]
*Oryza sativa*	Rice	O_2_^−^, H_2_O_2_	80–200 µM	[34,35,36,37]
*Amaranthus viridis* & *Amaranthus cruentus*	Green & Blood amaranth	O_2_^−^, H_2_O_2_	50 µM	[38]
*Chenopodium quinoa*	Quinoa	H_2_O_2_	5 mM Cr(III)	[39]
*Cucumis sativus*	Cucumber	O_2_^−^, H_2_O_2_	200 µM	[40]
*Brassica napus*	oilseed rape	O_2_^−^, H_2_O_2_, OH^−^	400 μM	[41,42]
*Brassica campestris*	Cabbage	O_2_^−^	1 mg/L	[43]
*Pisum sativum*	Pea	O_2_^−^, H_2_O_2_	100 μM	[44]
*Allium cepa*	Onion	O_2_^−^, H_2_O_2_, OH^−^	200 µM	[45]
*Matricaria chamomilla*	Chamomile	H_2_O_2_	120 µM Cr(III)	[46]
*Lens culinaris*	Lentil	H_2_O	250 µM	[47]
*Raphanus sativus*	Radish	O_2_^−^, H_2_O_2_	1.2 mM	[48]
*Pistia Stratiotes*	Lettuce	H_2_O_2_	10 mM	[49]

**Table 2:** Chromium-modulated antioxidant enzymes in various plant species. Ascorbate peroxidase (APX), catalase (CAT, dehydroascorbate reductase (DHAR), glutathione peroxidase (GPX), glutathione reductase (GR), glutathione S-transferase (GST), monodehydroascorbate reductase (MDHAR), peroxidase (POD), and superoxide dismutase (SOD).

Plant Species	Common Name	Enzymes	Cr(VI)	References
*Helianthus annuus*	Sunflower	CAT, SOD, POD, APX	20 mg/kg	[25,26]
*Triticum aestivum* *Hordeum vulgare*	Wheat & Barley	CAT, APX	22 mg/kg	[59]
*Brassica oleracea*	Cauliflower	CAT, SOD, POD	200 μM	[60]
*Pennisetum alopecuroides*	Fountain Grass	CAT, SOD, POD	1500 mg/kg	[61]
*Sorghum bicolor*	Sorghum	CAT, SOD, APX, GR, GST	64 ppm	[62]
*Brassica juncea*	Indian Mustard	GR, GPX, CAT, SOD, POD, APX, MDHAR, DHAR	300–500 μM	[17,63]
*Solanum melongena*	Eggplant	APX, GST, GR	25 µM	[64]
*Amaranthus viridis* & *Amaranthus cruentus*	Green & Blood Amaranth	CAT, SOD, POD, GST	50 μM	[38]
*Zea mays*	Maize	APX, CAT, SOD, POD	100–250 μM	[65,66]
*Hibiscus cannabinus*	Kenaf	CAT, SOD, POD	1.5 Mm Cr(III)	[67]
*Oryza sativa*	Rice	APX, CAT, SOD, POD, GR	20–100 μM	[68,69]
*Vigna radiate*	Mung Bean	CAT, SOD, POD	500 μM	[70]
*Brassica chinensis*	Pakchoi	CAT, SOD, POD	100 μM & 200 mg/kg	[71,72]
*Setaria italic*	Foxtail Millet	CAT, SOD, POD, APX	1000 μM	[73]
*Solanum nigrum* & *Parthenium hysterophorus*	Black Nightshade & Santa-maria	SOD, POD	500 μM Cr(III)	[74]
*Brassica rapa*	Turnip	SOD, APX	250 µM	[75]
*Brassica napus*	Rapeseed	CAT, SOD, POD, APX	500 μM	[76]
*Brassica campestris*	Cabbage	SOD, POD	1 mg/L	[43]
*Gossypium hirsutum*	Cotton	CAT, SOD, POD, APX	100 μM	[77]
*Corchorus olitorius*	Tossa Jute	CAT, SOD, POD, APX, GR	400 mg/kg	[78]
*Brassica napus*	Canola	CAT, SOD, POD, APX	50 μM	[79]
*Raphanus sativus*	Radish	CAT, SOD, POD	8 mM	[80]
*Hordeum vulgare*	Barley	CAT, SOD, POD, APX	225 μM	[81]

**Table 3:** Chromium-induced lipid peroxidation indicators in various plant species. Thio-barbituric acid reactive substances (TBARS) and malondialdehyde (MDA).

Plant Species	Common Name	LPO	Cr(VI)	References
*Arabidopsis thaliana*	Arabidopsis	MDA	400 µM	[8]
*Zea mays*	Maize	MDA	100–300 µM	[27,28,31,32,65]
*Triticum aestivum* *Hordeum vulgare*	Wheat & Barley	MDA	22 mg/kg	[59]
*Solanum lycopersicum*	Tomatoes	MDA	24.66 mg/k	[83]
*Oryza sativa*	Rice	MDA, TBARS	20–200 µM & 20 mg/L	[35,36,69,82,84,85]
*Limnobium laevigatum*	Floating Plant	MDA	70 µg/L Cr(III)	[86]
*Citrus reticulata Blanco*	Kinnow	MDA	750 µM	[87]
*Sorghum bicolor*	Sorghum	MDA	64 ppm	[62]
*Helianthus annuus*	Sunflower	MDA	20 mg/kg	[25]
*Brassica juncea*	Indian Mustard	MDA	100–500 μM & 100 mg/Kg	[17,63,88,89]
*Solanum melongena*	Eggplant	MDA	25 µM	[64]
*Tradescantia pallida*	Rose	MDA	20 mg/L	[90]
*Amaranthus viridis* & *Amaranthus cruentus*	Green & Blood Amaranth	MDA	50 μM	[38]
*Pteris vittata*	Chinese Brake	TBARS	5 mM	[91]
*Chenopodium quinoa*	Quinoa	MDA	5 mM Cr(III)	[39]
*Saccharum spp. Hybrid*	Sugarcane	MDA	50 ppm	[92]
*Cucumis sativus*	Cucumber	MDA	200 µM	[40]
*Pisum sativum*	Pea	MDA	100 μM	[44]
*Brassica rapa*	Turnip	MDA	250 µM Cr(III)	[75]
*Brassica napus*	Canola	MDA	50–100 μM	[79,93,94]
*Brassica oleracea*	Cauliflower	MDA	250 μM	[95]
*Salvinia minima*	Floating Fern	MDA	20 mg/L	[96]
*Tradescantia pallida*	Wandering Jew	TBARS	20 mg/L	[97]
*Gossypium hirsutum*	Cotton	MDA	100 μM	[77]
*Triticum aestivum*	Wheat	TBARS	200 μM	[98]
*Allium cepa*	Onion	MDA	200 μM	[45]
*Raphanus sativus*	Radish	MDA	125 m	[80]
*Miscanthus sinensis*	Chinese Reed	MDA	1000 μM	[99]
*Brassica napus*	Rapeseed	TBARS	480 μM Cr(III)	[100]

**Table 4:** Chromium-induced genotoxicity in various plant species.

Plant Species	Common Name	Genotoxicity	Cr- Type	References
*Glycine max*	Soybean	DNA damage	Cr(VI)/(III)	[22]
*Vicia faba*	Faba Bean	Micronucleus, Chromosomal fragmentation & bridging, Increase in % tail DNA, tail moment and Tail length	Tannery solid waste & Cr(VI)	[124,125,126,127]
*Allium cepa*	Onion	DNA damage, Chromosomal Aberrations, Micronuclei, Chromosomal fragmentation & bridging	Tannery solid waste, Tannery effluent & Cr(VI)	[45,125,127,128,129]
*Hordeum vulgare*	Barley	Chromosomal aberrations	Cr(VI)	[130]
*Vicia sativa*	Vetch	Chromosomal aberration, Chromosomal fragmentation & bridging	Wastes, Cr(VI)/(III)	[125,127,131]
*Raphanus sativus*	Radish	Chromosomal aberration	Cr(VI)/(III)	[125]
*Zea mays*	Maize	Chromosomal aberration	Cr(VI)/(III)	[125]
*Brassica napus*	Oilseed Rape	Methylation changes, mutation	Cr(VI)	[127,132]
*Arabidopsis thaliana*	Arabidopsis	DNA mutation	Cr(VI)	[127,133]

**Table 5:** Chromium-induced ultra-structure variation in numerous plant species. Epi-C-wax (epicuticular wax), TRICH (trichome), CW (cell wall), MITO (mitochondria), CM (cell membrane), THY (thylakoid), THY-O (thylakoid orientation), PG (plastoglobuli), SG (starch grains), GB (Golgi bodies), ER (endoplasmic reticulum), CHLP (chloroplast), I-cristae (interior- Cristae), T-nuclei (tubular nuclei), T-stroma (translucent stroma), ML (middle lamella), NM (nuclear membrane), and PT (Plant tissue used).

Plant Species	Common Names	PT	Effect	Cr-Type	References
*Glycine max*	Soybean	*L*	Loss of Epi-C-wax increased TRICH-number	Cr(VI)/(III)	[22]
*Brassica napus*	Oilseed rape	*L* & *R*	Alteration in CW, MITO, CM, THY, PG, SG, GB, ER, Irregular nucleus, THY disappeared, Increased SG number/size.	Cr(VI)	[41,42,120,134]
*Triticum aestivum* *Hordeum vulgare*	Wheat & Barley	*L*	Damaged CHLP, THY; Increased PG, Swollen MITO; altered I-cristae	Cr(VI)	[59]
*Nicotiana tabacum*	Tobacco	*L* & *R*	CW/CM not distinguishable, Disarranged CHLP structure, Undeveloped nucleus, damaged NM, Swelled/distorted THY, Damaged CHLP, MITO, Altered THY-O, Increased PG, Large SG	Cr(VI)	[122,123]
*Oryza sativa*	Rice	*L*	Swollen CHLP, grana/stroma/lamellae, Reduced grana/CHLP, Increased SG, Matrix zone expanded.	Cr(VI)	[35,135]
*Arabidopsis thaliana*	Arabidopsis	*R*	T-nuclei, GB disintegrated, spherical MITO, plastids; T-stroma; damaged MIOT, plastids; increased SG, amorphous material deposition in CW, ML, vacuoles, collapsed vacuoles, cytoplasm contained opaque lipid,	Cr(VI)	[23,47,136]
*Eichhornia crassipes*	Water Hyacinth	*L*	Damaged THY, MITO, CHLP (structure/distribution), grana	Cr(VI)	[137]
*Salvinia minima*	Floating Fern	*L*	Damaged CHLP, grana, THY, increased number/size of SG; large PG	Cr(VI)	[96]
*Taraxacum officinale*	Dandelion	*C*	Altered MITO with no/reduced I-cristae	Cr(VI)	[138]
*Hordeum vulgare*	Barley	*L* & *R*	Swollen CHLP, increased PG, Disintegrated/disappeared THY, MITO, Increased SG size/number, Increased vacuolar size, Cr-presence in CW, Vacuoles, Nucleus disruption/disappearance	Cr(VI)	[139]
*Solanum lycopersicum*	Tomatoes	*P*	Abnormal shaped reduced grana/CHLP; altered THY, MITO; reduced cristae numbers	Cr(III)	[140]
*Potamogeton crispus*	Curled Pondweed	*L*	Swollen CHLP, CHLP- envelop breakage, decreasing cristae, MITO vacuolization	Cr(VI)	[141]

**Table 6:** Chromium-induced alteration in photosynthesis and photosynthetic apparatus in various plant species. Chl a (Chlorophyll a), Chl b (Chlorophyll b), Chl t (total chlorophyll), Chl f (chlorophyll fluorescence), Trmmol (transpiration rate), Cond (stomatal conductance), photo (photosynthetic rate), PSII (photosystem II), Ci (intercellular CO_2_), *Φ_PSII_* (effective quantum of yield of photosystem II), qP (photochemical quenching), NPQ (non-photochemical quenching), P_N_ (net CO_2_ assimilation rate), ETR (electron transportation rate), pigment (photosynthetic pigments).

Plant Species	Common Name	Alteration in Photosynthetic Parameters	Cr(VI)	References
*Arabidopsis thaliana* & *Brassica juncea*	Arabidopsis & Indian Mustard	Reduced chl a, b, and t Reduced chl a, Reduced Chl t, Carotenoids, and net photo, b, and t, Gas exchange	400 µM 100–300 µM & 100 mg/Kg	[8] & [58,88,89,157]
*Helianthus annuus*	Sunflower	Reduced chl a, b, t, gas exchange, and carotenoid levels	Tannery effluent & 20 mg/kg	[26,158]
*Citrus reticulate*	Kinnow Mandarin	Decreased chl t, photosynthetic activity, Trmmol, Cond, and water use efficiency	0.75 mM	[87]
*Cyperus alternifolius* & *Coix lacryma-jobi*	Umbrella Palm & Adlay Millet	Inhibition in photosynthetic capacities	40 mg/L	[159]
*Solanum melongena*	Eggplant	Reduced pigments, photo, photochemistry of PSII	25 μM	[64]
*Oryza sativa*	Rice	Reduced Chl a, b, and carotenoids, Reduced F_v_/F_m_	80–200 µM	[34,35]
*Zea mays*	Maize	Reduced carotenoids, chl a, b, and t, Photo, Trmmol, Ci, Water use efficiency and intrinsic, Alteration in F_v_/F_m_, F_v_/F_0_, F_m_/F_0,_ and qP	Tannery effluent & 150–250 μM	[29,147,160]
*Amaranthus viridis* & *Amaranthus cruentus*	Green & Blood Amaranth	Inhibition photochemistry of PSII	50 μM	[38]
*Nicotiana tabacum*	Tobacco	Reduced Chl a, b, carotenoids, photo, gas exchange, Fv/Fm fluorescence	50 μM	[122]
*Sesbania grandiflora*	Hummingbird Tree	Reduced Chl t	1.92 mM/Kg	[161]
*Lactuca sativa*	Lettuce	Decreased levels Chl a, *Φ_PSII_*, qp, NPQ, P_N_ and RuBisCO activity	200 mg/L	[162]
*Triticum aestivum*	Wheat	Decline active reaction centers of PSII, ETR, and PSII heterogeneity	300 μM	[163]
*Humulus scandens*	Asian Hop	Decreased chl f parameters, chl t, and PSII reaction	300 mg/kg Cr(III)	[164]
*Cucumis sativus*	Cucumber	Decline in Fm, Fv, Fv/Fm, Fm/F0, and Fv/F0	200 µM	[40]
*Lemna minor*	Duckweed	Decreased in Fv/Fm, chl b	6 mg/L	[165]
*Pisum sativum*	Pea	Decreased pigments and Fv/Fm, Fv/F0 and qP, and NPQ increased	100 μM	[44]
*Raphanus sativus, Solanum lycopersicum* & *Spinacia oleracea*	Radish, Tomato & Spinach	Reduced photosynthetic activity and Chl t	100 mg/kg	[166]
*Brassica napus*	Rapeseed	Reduced chl t, and carotenoid	500 μM	[76]
*Solanum lycopersicum* & *Solanum melongena*	Tomato & Eggplant	Reduced pigments	7.5 ppm	[155]
